# An efficient incremental learning mechanism for tracking concept drift in spam filtering

**DOI:** 10.1371/journal.pone.0171518

**Published:** 2017-02-09

**Authors:** Jyh-Jian Sheu, Ko-Tsung Chu, Nien-Feng Li, Cheng-Chi Lee

**Affiliations:** 1 College of Communication National Chengchi University Taipei, Taiwan, R.O.C; 2 Department of Finance Minghsin University of Science and Technology Hsinchu, Taiwan, R.O.C; 3 Yilan County Government Police Bureau Yilan, Taiwan, R.O.C; 4 Department of Library and Information Science Fu Jen Catholic University New Taipei, Taiwan, R.O.C; 5 Department of Photonics and Communication Engineering Asia University Taichung, Taiwan, R.O.C; King Saud University, SAUDI ARABIA

## Abstract

This research manages in-depth analysis on the knowledge about spams and expects to propose an efficient spam filtering method with the ability of adapting to the dynamic environment. We focus on the analysis of email’s header and apply decision tree data mining technique to look for the association rules about spams. Then, we propose an efficient systematic filtering method based on these association rules. Our systematic method has the following major advantages: (1) Checking only the header sections of emails, which is different from those spam filtering methods at present that have to analyze fully the email’s content. Meanwhile, the email filtering accuracy is expected to be enhanced. (2) Regarding the solution to the problem of concept drift, we propose a window-based technique to estimate for the condition of concept drift for each unknown email, which will help our filtering method in recognizing the occurrence of spam. (3) We propose an incremental learning mechanism for our filtering method to strengthen the ability of adapting to the dynamic environment.

## Introduction

The study of spam filtering is an important research subject of information and communication management. At present, there is a wide variety of choices regarding filtering techniques to block spam email. James and Ray roughly divided them into two categories [1]: (1) non-machine learning, and (2) machine learning. The first category involves techniques such as Blackhole and Whitehole Lists, Digital Signature, Traffic Analysis, and son on [2–4], mainly by empirical experience, record the relevant spam rules for the recognition of unknown email. The benefit of such techniques is that it does not require a lot of computation. However, such techniques are prone to be penetrated and require frequent updates of lists and rules. The second category is to collect existing data, denoted as “training data”, and choose useful attribute fields of the data to generate meaningful rules or models, which will be applied to predict the newly arrived data [5–7]. However, spam emails cannot be effectively blocked by applying simply suchlike because the spammers continue to enhance their technologies to avoid filtering mechanisms and develop the cleverer spams that can evade the filter. A feasible solution is to generate rules or models by recollecting new data and relearning. However, the relearning method is both time consuming and costly. Another solution is the machine learning method without training process, such as the K-Nearest Neighborhood, Lazy learning Algorithm, Bayesian Classifier, and Case-based Reasoning [8–12]. As this type of techniques has not clearly constructed knowledge structure while handling data, it only needs to record these data and their attributes. When unknown values appear in the attribute fields of the newly arrived data, the newly arrived data will be simply classified by computing similarity between it and other data. However, this method is time consuming in classification. Larger volume of data will lead to more time consumed.

Moreover, most of spam filters of machine learning are designed to screen out spams in static environment. Nevertheless, the Internet is a dynamic database and will generate constantly the problem of “concept drift”, which indicate that conceptual model of data changes along with time [13–19]. For example, the survey of spam types conducted by Symantec categorized spam emails prior to September 2008 into 8 types. However, in the survey report dated October 2008, there were 9 types of spam emails with an additional type named “Political” [20]. The explanation of the additional type by Symantec was as follows: as the US was in the election season, the election had become an issue of concern, hence, spam mail titles had been changed into themes related to the election. For example, some spammers sent out a large number of spam emails with political headlines under the disguise of news of the election candidate (Obama) to lure receivers to read. However, it is costly and time-consuming to reconstruct the filtration models or rules of machine learning filter to address concept drift. Therefore, most of current machine learning spam filters have some shortcomings in dealing with concept drift.

Spam prevention has been becoming the critical issue of Internet applications and information communication for many years. There are numerous methods in present research literatures with respect to recognition of spam emails. For instance, in 1996, Cohen proposed a filtering method based on RIPPER learning algorithm [21]; in 1999, Drucker *et al*. applied Support Vector Machines (SVMs) to classify spam emails and non-spam emails [22]; in 2001, Carreras and Marquez introduced a new method based on AdaBoost algorithm[23]; in 2005, Delany and Cunningham presented a kind of KNN (the *k* nearest neighbors) method [13]; in 2007, He and Bo filtered spams by constructing a new asymmetric boosting method, Boosting with Different Costs [24]; and in 2008, Hsiao and Chang constructed an incremental clustering-based filtering technique [25]; in 2012, DeBarr and Wechsler proposed two alternative methods of random projections and compared their performance for robust and efficient spam detection when trained using a small number of examples [26]; in 2014, Zhou *et al*. proposed a cost-sensitive three-way spam filtering method [12]. Moreover, Khan *et al*. recently suggested some intelligent methods such as evolutionary algorithms to fight against spam botnets [27–29].

Most of the filtering methods mentioned above were designed to intercept spams by scanning fully the content of email. However, the manner of examining fully email’s content would consume a lot of time. In 2007, Wang and Chen proposed a method by using header session [30]. Then, in 2009 Sheu and Chu developed an efficient filtering method, which analyzed only email’s header section without scanning fully the content of email [31]. In 2013, Liu *et al*. extracted attributes from all possible forged header fields to expand the feature sets, then used the rough set theory to classify the sample sets [32].

This study aims to address the above issues by designing a spam filtering mechanism based on machine learning method, in order to solve the problem of concept drift concurrently. We will apply the uncomplicated decision tree data mining algorithm to learn association rules about spams from training emails. Based on these rules, we will propose a more efficient spam filtering mechanism with the following major advantages:

1. We check only the header of email in order to improve executive efficiency and diminish the computation complexity, which is different from those spam filtering methods at present that have to analyze fully the email’s content.
2. Regarding the solution to the problem of concept drift, we propose a window-based technique to estimate for the condition of concept drift for keywords found recently in spam emails. It will help our filtering method in recognizing the occurrence of spam.
3. We propose an incremental learning mechanism for our filtering method to strengthen the ability of adapting to the dynamic environment.

The remainder of this paper is organized as follows. Section 2 discusses the related background of this paper. In Section 3, we propose a new spam filtering method based on decision tree data mining technique. The experimental results of our method are shown in Section 4. Section 5 concludes this paper.

## Related background

### Concept drift

In reality, the concept of data changes along with time. The continuous change of data along with time would result in the renewal of conceptual model, causing the problem of concept drift [19]. Let the data stream be represented by consecutive blocks. In stable status, the distribution of data points within the successive blocks tends to be similar, then “concept stable” occurs in this case. If the distribution of data points varies in the adjacent blocks, “concept drift” may occur. Considering a given block, if its distribution of data points is contrary to that of adjacent block, “concept shift” may occur.

According to recent studies, classification methods to address the concept drift problem can be divided into the following categories:

1. **Instance selection:** the purpose is to filter as much as possible data instances most related to the current concept. The most common technology to address the concept drift problem is the Window-based method [9, 16–17], which stores the recent data instances in the window with limited capacity. When new data instances are added and the window is filled up, the old data instances in the window will have to be removed. This method is used to record the current data instances, by which the occurrence of concept drift of future data instances will be predicted.
2. **Instance weighting:** the method of instance weighting is to assign different weights to each data instance according to the access time. Each data instance is assigned a weight according to its arrival time. The classifier obtains the classification results by applying computation related with weights of data instances. And the data instance should be disposed if it exceeds the predetermined time scope [33].
3. **Ensemble learning:** this method is to use more than two classifiers to determine the result by weighted voting. In case of various circumstances, different classifiers are used to predict the classification results and assigned different weights. Combine these classifiers and select the optimal combinations to find out the best classification results to solve the concept drift problem [9, 13].

### Decision tree algorithm

Decision tree algorithm is the data mining technique upon the tree data structure. The usual statistical method can only calculate the distribution of the surface of data whereas decision tree algorithm can detect the potential association rules of data. Moreover, the association rules can be applied to predict or classify the unknown data.

ID3 (Iterative Dichotomiser 3) and C4.5 are two most well-known methods among the decision tree data mining algorithms [34–36]. ID3 only supports categorical attributes. However, C4.5 (an improved method of ID3) can support both categorical attributes and numerical attributes simultaneously. Since the data attributes adopted in this research are the mixture of numerical type and categorical type, we select C4.5 as the data mining algorithm applied in this paper.

Decision tree is constructed from top node (the “root”) to bottom nodes (the “leaves”). Let “*Target Attribute*” be the attribute which is concerned objective of our research. For example, the attribute “*email type*” whose value is either “spammy” or “legitimate” is the Target Attribute of this study. And let “*Critical Attributes*” be the other attributes which interest us in this research.

In the beginning, all data instances are stored in the root node. The C4.5 algorithm will select a Critical Attribute with the maximum *Gain Ratio* but not yet selected, and divide all data instances into child nodes according to their values of the selected Critical Attribute. Then, each child node repeats the above-mentioned procedures of selecting Critical Attribute, until reaches any of the stop conditions, to complete building procedures of decision tree. Note that C4.5 algorithm has some stop conditions as follows: 1) Target Attribute’s values of all data instances in this child node are exactly the same; 2) all Critical Attributes have been selected; 3) the number of data instances in this child node is less than a specific number *M* which is defined in advance.

Now we discuss the formulas about the *Gain Ratio* [35–36]. Given a certain attribute *F* and a data instance set *C*, the *Information Gain* of attribute *F*, denoted by *Gain*(*F*), will concern the *Entropy* of *C*, denoted by *E*(*C*), which is computed by the following formula:

$$E(C)=-\sum_{i=1}^{t}\frac{p_{i}}{n}\times \log_{2}\frac{p_{i}}{n}$$

where *t* is the number of Target Attribute’s values, *p*_*i*_ is the number of data instances corresponding to the *i*-th value of the Target Attribute in *C*, and *n* is the number of data instances in *C*. Then *Gain*(*F*) is calculated by the following formulas:

$$Gain(F)=E(C)\text{-}E^{+}(F)$$

$$E^{+}(F)=\sum_{i=1}^{k}(n_{i}/n)\times E(C_{i})$$

where *k* is the number of values of attribute *F*, *C*_*i*_ is a subset of *C* including the data instances corresponding to the *i*-th value of attribute *F*, and *n*_*i*_ is the number of data instances in *C*_*i*_. Then, *Gain Ratio* of *F*, denoted by *GainRatio*(*F*), can be calculated by the following formulas:

$$Gain\;Ratio(F)=\frac{Gain(F)}{SplitInfo(F)};$$

$$SplitInfo\left(F\right)=-\overset{k}{\underset{i=1}{\sum }}\frac{n_{i}}{n}\log_{2}\frac{n_{i}}{n}.$$

Now we introduce the detailed procedure of C4.5 algorithm as follows [35–36].

**Step 0.** All data instances are stored in the root node, select a Critical Attribute, say *F*, with maximum *GainRatio*(*F*). Based on the values of this Critical Attribute, branch child nodes and distribute the data instances with the identical value of attribute *F* into the same child node. Then the following steps will be executed for each child node.
**Step 1.** If all data instances in this child node have the same value of Target Attribute, say *f*_*i*_, this child node will be marked as a leaf node. Then this leaf node is labeled as *f*_*i*_, and the building process of this child node is completed.
**Step 2.** If all Critical Attributes have been selected, then this child node cannot be further split. Mark this child node as a leaf node. But data instances in this child node do not necessarily have the same value of Target Attribute. Therefore, the value of Target Attribute possessed by the majority of data instances is selected, say *f*_*j*_. Then this leaf node is labeled as *f*_*j*_, and the building process of this node is completed.
**Step 3.** If the number of data instances in this child node is less than *M*, the building process of this child node will be stopped. We mark this child node as a leaf node. Select the value of Target Attribute possessed by the majority of data instances, say *f*_*k*_. Then this leaf node is labeled as *f*_*k*_, and the building process of this node is completed.
**Step 4.** Calculate the *Gain Ratio* for each Critical Attribute which is not selected yet. And choose the Critical Attribute, say *F*, with the maximum *Gain Ratio*. Based on the values of attribute *F*, branch corresponding child nodes downward and divide the data instances with the identical value of attribute *F* into the same child node.
**Step 5.** Step 1~Step 5 are repeated for each of the child nodes generated in Step 4 respectively.

In the decision tree constructed at the end, each leaf node will be labeled as a value of the Target Attribute. Each path from root node to leaf node will form an association rule. All internal nodes on the path constructed a row of “if” judgment of Critical Attributes. With the “then” result presented by the labeled value of the leaf node, there is the association rule of “if-then” pattern constructed.

Then we will calculate “*degree of purity*” and “*degree of support*” for each leaf node. Let the labeled value of leaf node *C* be *Label*(*C*), and |*Label*(*C*)| be the number of data instances whose Target Attribute’s value is equal to *Label*(*C*) in *C*. Moreover, let *Purity*(*C*) denote the degree of purity of *C*, and *Support*(*C*) denote the degree of support of *C*, respectively. Then *Purity*(*C*) and *Support*(*C*) are defined by the following formulas:

Purity(C)=(|Label(C)|/|C|)*100%;

Support(C)=(|C|/N)*100%;

where the number of data instances in node *C* is denoted by |*C*|, and *N* is the number of total data instances in the root node.

## Research architecture

In this research we don’t check the content of email in order to diminish the computation complexity. We will focus on analysis of email header’s basic attributes such as email title, sender’s name, sender’s email address, sending date. Then, we apply decision tree data mining technique to look for the association rules about spam emails. Finally, we propose a systematic method based on these association rules to accurately identify an unknown email to be either legitimate or spammy.

Before the execution of our email filtering method, the “Keyword Database” will be built in advance. In our email filtering method, the Keyword Database is composed of two kinds of keyword table:

1. Spam Keyword Table: This table records those suspicious keywords found frequently in spam emails. In this study, we will take the spam keywords table proposed by Sanpakdee et al [37]. as the original content of our Spam Keyword Table.
2. Legitimate Keyword Table: This table records keywords commonly found in legitimate emails and seldom discovered in spams.

**Fig 1:**
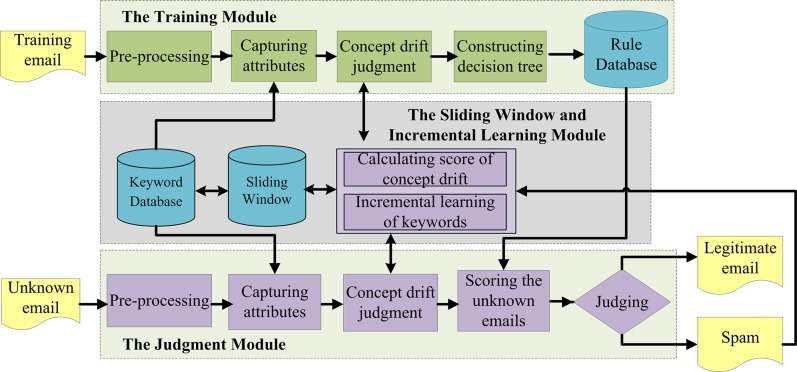
The architecture of our email filtering method.

Now we introduce the architecture of our email filtering method. As shown in Fig 1, the architecture of our email filtering method is divided into the following three major modules:

1. The Training Module: The purpose of this module is to seek for association rules between the Target Attribute (spammy or legitimate) and Critical Attributes of training emails, which will be applied to classify unknown emails in the Judgment Module.
2. The Sliding Window and Incremental Learning Module: This module will use a window-based technique to calculate the score of concept drift for each email to estimate for the condition of concept drift in this email. Moreover, it will continuously learn the newly arrived spam keywords into the Keyword Database.
3. The Judgment Module: This module is the core of our spam filtering method, it will classify each unknown email to be either a legitimate email or a spam.

### The training module

In the Training Module, the emails collected in advance are taken as the training data. We set the attribute “email type” to be the Target Attribute. If the training email is spammy, then the email type is labeled as “spammy”, if it is legitimate, then the email type is labeled as “legitimate”. Moreover, as shown in Table 1, eleven “Critical Attributes” are defined according to the important fields of email header including 9 Critical Attributes of binary values and 2 Critical Attributes of numerical values. We will apply the C4.5 decision tree algorithm to analyze the associative rules between the 11 Critical Attributes and the Target Attribute by using these training emails.

**Table 1:** The 11 Critical Attributes.

Category	Attribute ID	Critical Attribute	Value
**Sender**	***A***	Length of sender’s name is abnormal	If the length of sender’s name is more than 9 characters, it is set at 1 (True), otherwise 0 (False)
	***B***	Spam keyword is found in sender’s name	If any spam keyword is found, it is set at 1 (True), otherwise 0 (False)
	***C***	Spam keyword is found in sender’s address	If any spam keyword is found, it is set at 1 (True), otherwise 0 (False)
	***D***	Similar words is found in sender’s name and email’s title	If any similar word found in sender’s name and email’s title, it is set at 1 (True), otherwise 0 (False)
**Title**	***E***	Wrong word in email’s title is found	If any wrong word is found in email’s title, it is set at 1 (True), otherwise 0 (False)
	***F***	Email’s title has one spam keyword	If email’s title has only one spam keyword, it is set at 1 (True), otherwise 0 (False)
	***G***	Email’s title has two spam keywords	If email’s title has two spam keywords, it is set at 1 (True), otherwise 0 (False)
	***H***	Email’s title has more than two spam keywords	If email’s title has more than two spam keywords, it is set at 1 (True), otherwise 0 (False)
**Time and size**	***I***	Sending date and receiving date are abnormal	If the date of sending distinctly differs from the date of receiving, it is set at 1 (True), otherwise 0 (False)
	***J***	Email’s size	The size of email is recorded in the unit of byte
**Concept drift**	***K***	The score of concept drift	Calculated by the Sliding Window and Incremental Learning Module.

The detailed process of the Training Module is described by the following stages.

#### Stage 1. Pre-processing

In this stage, the header section of each training email will be processed. First, remove the meaningless stop words from the training email’s header section. Then, apply the Porter stemming algorithm [38] to strip suffixes from English words. Thus, the noise in fields of email’s header section can be reduced.

#### Stage 2. Capturing attributes

Then, each training email will be checked to capture the values of all necessary attributes, which will be used for applying the C4.5 algorithm. For each training email, the Target Attribute’s value depends on its type (“spammy” means this email is a spam; “legitimate” means the opposite situation). Moreover, the 11 Critical Attributes are defined as shown in Table 1, whose values are decided by checking the corresponding fields of header section and looking up the Keyword Database (if necessary). These 11 Critical Attributes are divided into four categories of “Sender”, “Title”, “Time and size”, and “Concept drift”.

The attributes labeled *A*~*D* of “Sender” investigate the statuses of related fields of email’s sender. The attributes labeled *E*~*H* of “Title” analyze the related fields of email’s title. The attributes labeled *I*~*J* of “Time and size” investigate whether email’s sending date and receiving date are abnormal and record email’s size. The attributes labeled *K* of “Concept drift” contain the score of concept drift, which is calculated by the Sliding Window and Incremental Learning Module. Note that the attributes labeled *A~I* are data of binary values while attributes labeled *J*, *K* are data of consecutive values.

#### Stage 3. Concept drift judgment

In this stage, all keywords found in email’s title are delivered into the Sliding Window and Incremental Learning Module to calculate the score of concept drift of this email.

#### Stage 4. Constructing decision tree and scoring rules

This stage employs the decision tree algorithm C4.5 to construct the decision tree, which will bring out the potential association rules of “if-then” pattern between the 11 Critical Attributes and the Target Attribute (“spammy” or “legitimate”). Then we will score each rule by using formulas based on the values of its degree of support and degree of purity.

Given an association rule *R*, we assume that the associated leaf node is *C*. Moreover, let *m* be the number of emails whose the Target Attribute’s value is “spammy” in node *C*. Before describing the scoring formula for the rules, we will introduce four important functions: *SpamDegree*(*R*), *RuleSupport*(*R*), *W*(*RuleSupport*(*R*)), and *S*(*RuleSupport*(*R*)).

The spam degree function *SpamDegree*(*R*) implies rule’s “intensity” to classify emails as spams. It is defined as follows:

SpamDegree(R)=Purity(C)ifLabel(C)="spammy";

$$\text{and}\;SpamDegree(R)=(\frac{m}{\left|C\right|})*100\%\;otherwise,$$

where |*C*| is the number of emails contained in node *C*. Moreover, *RuleSupport*(*R*) records the degree of support of rule *R*, and we define its value as follows:

RuleSupport(R)=Support(C).

Now, we can compute the values of degree of support for all rules, and then we assume that *Support*_*MAX*_ is the maximum one and *Support*_*MIN*_ is the minimum one. The function *W*(*RuleSupport*(*R*)) records the weighted value of *RuleSupport*(*R*), which is described as follows:

$$W(RuleSupport(R))=\frac{RuleSupport(R)}{Support_{MAX}+Support_{MIN}}\times 100\%.$$

Now the weighted values of rule support of all rules can be computed by above formula. Let *W*_*MAX*_ be the maximum one and *W*_*MIN*_ be the minimum one of all weighted values. Then, the function *S*(*RuleSupport*(*R*)) will calculate the score of *RuleSupport*(*R*), which is relative to the ranking of weighted value of rule *R*:

$$S(RuleSupport(R))=\frac{W(RuleSupport(R))-W_{MIN}}{W_{MAX}-W_{MIN}}\times 100\%.$$

Finally, the score of rule *R*, defined by *Score*(*R*), can be computed by the following formula:

Score(R)=(0.7×SpamDegree(R)+0.3×S(RuleSupport(R)))×100.

After computing the scores, all of the rules are stored into the Rule-Database, which will be accessed by the Judgment Module for classifying unknown emails.

We choose out the minimum rule’s score from the rules with spam degree’s value more than 80% (i.e., *SpamDegree*(*R*)≥80%), and set it as the threshold *t* for judging whether an unknown email is a spam. We choose this threshold by performing a lot of experiments, and we found that the minimum rule’s score of the rules with spam degree’s value more than 80% is enough to classify spams correctly.

### The sliding window and incremental learning module

This module is in charge of the following two major tasks: (1) Calculating the score of concept drift for each training email of the Training Module or each unknown email of the Judgment Module; (2) Incremental learning of new keywords of spam emails recognized by the Judgment Module.

#### (1) Calculating the score of concept drift

The score of concept drift of each email will be calculated by checking all keywords contained in email’s title. This module uses a window-based technique to estimate for the condition of concept drift of keywords in this email. We construct a “Sliding Window” to keep the keywords found recently in spam email’s title. The size of the Sliding Window is assumed to be 30 days. In other words, the Sliding Window will store the spam keywords found in the past 30 days.

Assume that the email’s title includes *k* keywords which can be found in the Sliding Window, and let these keywords be denoted by *Keyword*(*i*) for 1*≤ i≤ k* where *k* is a positive integer. We suppose that the email’s sending date is denoted as *SendingDate*. The following definitions are necessary for calculating the score of concept drift for each email.

1. *DateDiffer*(*i*): This function will compute the sustained time since the latest date time that *Keyword*(*i*) appeared in an incoming email. Let *AccessDate*(*i*) record the latest date time that *Keyword*(*i*) was found in an incoming email. Then the function can be computed through the following formula:
  DateDiffer(i)=SendingDate−AccessDate(i).
2. *KeywordCount*(*i*):Because the spam keyword’s frequency is also a significant evaluative criterion of concept drift, we use this function to accumulate the found times of *Keyword*(*i*) since it was recorded into the Sliding Window. The value of *KeywordCount*(*i*) will increase by 1 whenever the same keyword, *Keyword*(*i*), is included in the incoming email’s title.
3. *TimeWeight*(*i*): This function will compute the weighted value of estimating the degree of concentrated appearance for each spam keyword. The concentrated appearance of a new spam keyword usually implies occurring of concept drift, which will result in the smaller value of *DateDiffer*(i). Thus, we use Sliding Window’s size, which is assumed to be 30 days, to compute weighted value. Given a spam keyword *Keyword*(*i*) with 1≤ *i*≤ *k*, the weighted value is calculated according to the following formula:
  TimeWeight(i)=30−DateDiffer(i).

Now the score of concept drift of this email, denoted as *ConceptDriftScore*, can be computed through the following formula.

$$ConceptDriftScore=\sum_{i=1}^{k}\left(KeywordCount(i)\times TimeWeight(i)\right).$$

Obviously, we can observe that this email will acquire a high score of concept drift if its spam keywords possess the higher frequencies and concentrated appearances.

#### (2) Incremental learning of spam keywords

We assume that all of the emails are entered into our filtering system in the increasing order of the sending date. Whenever a spam email is entered to the filtering system, the spam keywords contained in its title will be recorded into the Sliding Window or written into the Spam Keyword Table of Keyword Database if necessary. By applying this incremental learning method, the contents of Sliding Window and Keyword Database will be expanded progressively.

In our method, each keyword found in the title of spam emails (the spam training emails of the Training Module or the spam emails identified by the Judgment Module) will be considered a candidate for entering into the Sliding Window. If the keyword has been recorded in the Sliding Window, we add 1 to its frequency value (i.e., *KeywordCount*(*i*)). If it is not recorded in the Sliding Window, we have to judge whether it is a spam keyword or not by checking two simple tables (Legitimate Keyword Table and Spam Keyword Table). If this keyword has been recorded in the Spam Keyword Table, we insert it into the Sliding Window and initialize the values of its related functions. If this keyword is not recorded in the Spam Keyword Table, we will consider the following two cases:

1. The first case is that this keyword is listed in the Legitimate Keyword Table. It implies that this keyword is rightful and not related to the symptom of spam emails, hence we ignore it.
2. The second case is that this keyword is not listed in the Legitimate Keyword Table. In this case, this keyword can be treated as a new suspicious spam keyword. Therefore, we will insert it into the Sliding Window and initialize the values of its related functions. Moreover, this new suspicious spam keyword will be learned into the Spam Keyword Table simultaneously to strengthen the table’s ability in filtering the suspicious unknown emails.

**Fig 2:**
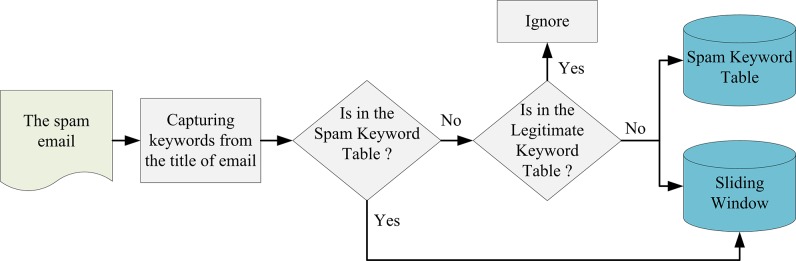
The incremental learning of spam keywords of the Sliding Window.

Note that the Sliding Window store the spam keywords found within the past 30 days in our method. Each spam keyword with the value of *DateDiffer*(*i*) more than 30 days will be deleted from the Sliding Window. The major process of incremental learning mechanism is described in Fig 2.

### The judgment module

The Judgment Module will judge the unknown email to be either a legitimate mail or a spam. First, pre-processing is carried out to reduce noise for each unknown email. Then, the attributes in email’s header can be captured precisely. According to the 11 Critical Attributes in Table 1, the associated Critical Attributes of each unknown email will be checked and recorded. Then, the score of concept drift of the unknown email is calculated by the Sliding Window and Incremental Learning Module. Finally, the Rule Database generated from the Training Module is applied to classify this unknown email. Moreover, if an unknown email is classified as a spam, then the keywords of its title will be sent to the Sliding Window and Incremental Learning Module to learn new keywords into the Sliding Windows and Keyword Database.

The detailed process of this module can be partitioned into the following stages:

#### Stage 1. Pre-processing

This “Pre-processing” stage is similar to that of the Training Module. When an unknown email arrived in our system, the noise in fields of header section will be reduced in this stage.

#### Stage 2. Capturing attributes

Like the stage of Capturing attributes in the Training Module, this stage will check each unknown email according to the 11 Critical Attributes established in Table 1, and the values of these associated Critical Attributes of unknown email will be recorded for further computations in the following stages.

#### Stage 3. Concept drift judgment

In this stage, we compute the score of concept drift for each unknown email to estimate for the condition of concept drift in this mail. All keywords found in email’s title are delivered into the Sliding Window and Incremental Learning Module to calculate the score of concept drift of this unknown email.

#### Stage 4. Scoring the unknown emails

Now, all the 11 Critical Attributes of this unknown email have been decided. According to these Critical Attributes’ values, the unknown email will dovetail with some association rule, say, *R* in the Rule-Database built in the Training Module. And we will define the score of the unknown email to be *Score*(*R*), which is calculated by the formula described in Stage 4 of the Training Module.

If any unknown email’s score is higher than the threshold *t* (as defined in Stage 4 of the Training Module for judging whether an unknown email is a spam), it will be regarded as a spam.

#### Stage 5. Incremental learning of new keywords of spam emails

If an unknown is identified as a spam, keywords found in its title should be considered candidates for learning into the Sliding Window and the Keyword Database. Then these keywords will be delivered to the Sliding Window and Incremental Learning Module to analyze further. The detailed analysis process of the Sliding Window and Incremental Learning Module is as described earlier.

## Experimental results

### Experiment design

In order to confirm the accuracy and efficiency of the spam filtering method proposed in this study, the following three experiments are designed:

- **Experiment A**: The first experiment is to test the accuracy of our spam filtering system without incremental learning mechanism and evaluation of concept drift. Note that the Critical Attribute “Concept drift”, which computes the score of concept drift, is removed in this experiment. Thus, we only apply 10 Critical Attributes to the calculation of the decision tree algorithm.
- **Experiment B**: The second one is to test the performance of the incremental learning mechanism (without the calculation of score of concept drift, however) proposed in this study, and whether there is any impact of the incrementally increasing of spam keywords on the accuracy of the spam filtering system. Note that the Critical Attribute “Concept drift” is removed in this experiment. We apply only 10 Critical Attributes to the decision tree algorithm.
- **Experiment C**: The third one is to evaluate whether the cooperation of two mechanisms: “Incremental learning” and “Weighted sliding window” proposed in this paper can effectively address the problem of spam concept drift and improve overall accuracy. This experiment is distinct from the Experiment B. In this experiment, the Critical Attribute “Concept drift” will be computed and inputted into the decision tree’s calculation, that is, we apply all of 11 Critical Attributes to the decision tree algorithm.

The experimental emails of this study are taken from the TREC (Text Retrieval Conference) Public Corpus [39]. The TREC email data set is commonly applied in research papers about spams, it is provided by a seminar jointly held by US NIST (National Institute of Standards And Technology) and DARPA (Intelligence Advanced Research Projects Activity) for information retrieval and relevant researchers. The data set collects 25220 legitimate emails, 50199 spam emails in a total of 75419 emails.

To evaluate the performance for our spam filtering method, we need some efficacy assessment indexes. This study employs the indexes: “*Precision*” and “*Recall*”, which are commonly used for document classification. Moreover, we calculate the harmonic mean “*F-measure*”. The decision confusion matrix, as shown in Table 2, is used to explain the calculation equations listed as follows [10, 13, 25]. Note that the four cases *A*, *B*, *C*, and *D* in Table 2 are all recorded by the number of emails.

**Table 2:** Four cases of judgment.

	Email’s categorization in reality	
	Spam	Legitimate mail
To be judged as spam	*A*	*B*
To be judged as legitimate mail	*C*	*D*

1. **Precision:** it refers to the ratio of the emails identified correctly in the emails judged as the certain category, representing filter’s capability to classify correctly such category of emails. This study calculates the “*Spam Precision*” from the perspective of identifying spams, and the “*Legitimate Precision*” from the perspective of identifying legitimate emails. Finally, it sets the value of “*Precision*” as the mean of Spam Precision and Legitimate Precision with equation listed as follows:
  $$Spam\;Precision=\frac{A}{A+B};$$
  $$Legitimate\;Precision=\frac{D}{C+D};$$
  $$Precision=\frac{Spam\;Precision+Legitimate\;Precision}{2}.$$
2. **Recall:** it calculates the ratio of the emails judged correctly. The “*Spam Recall*” is defined as the probability of judging correctly spammy emails as spams. And the “*Legitimate Recall*” is defined as the probability judging correctly legitimate emails. Finally, the “*Recall*” value is set as the mean of the Spam Recall and Legitimate Recall with equation listed as follows:
  $$SpamRecall=\frac{A}{A+C};$$
  $$LegitimateRecall=\frac{D}{B+D};$$
  $$Recall=\frac{Spam\;Recall+Legitimate\;Recall}{2}.$$
3. **F-measure:** the harmonic mean of the Precision and Recall with equation listed as follows:
  $$F-measure=\frac{2\times Precision\times Recall}{Precision+Recall}$$

This study first analyzes the sending time field of the emails in TREC to classify the emails on a monthly basis. If the sending time field of the email is abnormal (for example, no sending time or abnormal sending time), remove this email. From the 75419 emails in the same year of the TREC data set, this study obtains emails of valid sending time including 19057 legitimate emails and 40059 spam emails. According to the increasing order of the sending time, these emails can be classified into four categories: (1) April, (2) May, (3) June, and (4) July. To achieve the calculation fairness of the experiment, the ratio of legitimate emails and spam emails is set as 1:1 for each month. Then the emails of each month will be applied to the experiment sequentially. Moreover, we extract randomly 10% emails of each month as the training emails, which include 3816 emails and will be applied in the Training Module. Then the remaining emails are treated as the unknown emails, which will be applied in the Judgment Module. The email numbers of the four months are shown in Table 3.

**Table 3:** The email numbers of the four months.

Category	April	May	June	July	Total
Legitimate emails	4729	6511	6639	1202	19081
Spam emails	4729	6511	6639	1202	19081
Training emails	946	1302	1328	240	3816
Unknown emails	8512	11720	11950	2164	34346

### The analysis of experimental result

Now we discuss the results of three spam filtering experiments of this study as follows.

**(1) The analysis of Experiment A:** As shown in Table 4, in the absence of incremental learning, the four month average of F-measure is 0.8825, which is the minimum value among three experiments. Note that this experiment is to use only the spam keyword table proposed by Sanpakdee [37] to test the accuracy of the spam filtering system, and it does not apply any relearning mechanism. Due to the limited number of spam keywords in judging email, many keywords related to spam in email’s header section cannot be accurately identified in this experiment.

**Table 4:** The F-measure values of three experiments.

Month	Experiment A	Experiment B	Experiment C	Mail amount
April	0.874	0.906	0.936	8512
May	0.910	0.960	0.969	11720
June	0.888	0.888	0.966	11950
July	0.858	0.962	0.977	2164
**Average**	0.883	0.928	0.962	34346

**(2) The analysis of Experiment B:** The result of Experiment B is also shown in Table 4. By applying the incremental learning mechanism proposed in this paper, the monthly F-measure of the Experiment B can be found as increasing significantly after learning new keywords into our Spam Keyword Table. Compared with the Experiment A, the four month mean F-measure of this experiment is raised obviously to 0.928. As new keywords monthly collected by the Sliding Window can be learned into the Spam Keyword Table of the Keyword Database, more spam keywords are available for improving the efficiency of filtering spams.

**(3) The analysis of Experiment C:** With the assistance provided by the calculation of Critical Attribute “Concept drift”, the Experiment C has the highest F-measure at 0.972 in May. As shown in Table 4, the four month average F-measure is 0.9594, being higher than those of Experiment A and Experiment B. This method applied in the Experiment C can not only add new keywords to detect unknown spam emails by incremental learning, but also employ the score of concept drift (recorded in the Critical Attribute “Concept drift”) to help the decision tree algorithm to detect the suspicious titles frequently appear in recent days. Therefore, the values of F-measure in Experiment C are higher than those of the other experiments.

Table 5 shows the Precision, Recall and F-measure of various months by the spam filtering method proposed in this study. With the incremental learning mechanism, our filtering method’s ability of adapting to the dynamic environment has been strengthening. Obviously, the values of Precision, Recall and F-measure are increasing month after month. Our filtering method in July has Recall as high as 0.995, Precision at 0.960, and F-measure at 0.977. The average values of Precision, Recall, and F-measure of each month are 0.955, 0.970, and 0.9594 respectively. Note that we check only the header of email to improve executive efficiency and diminish the computation complexity. Therefore, our method is different from those filtering methods at present that have to analyze fully the email’s content.

**Table 5:** The detailed datum of the Experiment C.

**month**	**Precision**	**Recall**	**F-measure**
April	0.940	0.932	0.936
May	0.962	0.976	0.969
June	0.956	0.976	0.966
July	0.960	0.995	0.977
**Average**	**0.955**	**0.970**	**0.962**

Table 6 shows the top 5 keyword counts computed by the Sliding Window of each month. These keywords are extracted from the spam emails’ title. As seen, these spam keywords repeatedly appears during one month. And the keywords recorded by the Sliding Window during various months are not necessarily the same. The spammers always send out a large number of advertising spams with the similar titles, therefore, such type of emails can be detected easily by the mechanism of the weighted Sliding Window proposed in this paper. Relatively higher score of concept drift (i.e., the value of Critical Attribute “Concept drift”) will be judged for such type of emails to help our filtering system to correctly detect this type of spams.

**Table 6:** Top five keyword counts by month.

Ranking	April		May		June		July	
	drift keyword	count	drift keyword	count	drift keyword	count	drift keyword	count
**1**	pharmaci	260	approv	236	price	297	medic	262
**2**	viagra	211	pharmaci	186	medic	268	nation	184
**3**	pill	190	line	165	mhln	268	unit	183
**4**	ciali	177	fda	157	state	164	state	183
**5**	approv	171	photoshop	153	unit	161	associ	183

Table 7 shows the comparison of the method proposed in this paper and other methods. The four month average F-measure in this study is 0.962, which is better than that of each of other methods. The Precision values of some methods are higher than the 0.955 of our method, representing that those methods have higher spam classification accuracy than this study. However, their Recall values are all lower than the 0.97 of this study and our method has the best value of F-measure, indicating that the overall judgment accuracy rate of our filtering system in this study is relatively higher. Moreover, our method checks only email’s header section, and this technique will improve executive efficiency and diminish computation complexity.

**Table 7:** The comparison of different spam filtering methods.

Filtering method	Precision	Recall	F-measure	Checking the complete content of email
**The method proposed in this paper**	**0.955**	**0.970**	**0.962**	**No**
Lazy Learning Algorithm [15]	0.99	0.89	0.937	Yes
Case-based Reasoning [9]	0.92	0.96	0.94	Yes
SpamHunting [10]	0.99	0.86	0.92	Yes
Genetic Algorithm [37]	0.898	0.757	0.82	Yes
ICBC: Incremental clustering-based classification [25]	0.958	0.961	0.959	Yes

## Conclusions and future studies

This research has proposed an efficient spam filtering method based on machine learning and decision tree data mining techniques, analyzed the association rules among varied characteristics of spam email’s header section and applied these rules to develop an efficient and accurate spam filtering mechanism. Our spam filtering method is provided with the following major advantages: (1) Checking only email’s header section in order to improve executive efficiency and diminish computation complexity, which is different from those spam filtering methods at present that have to analyze fully email’s content. (2) We constructed the weighted “Sliding Window” to calculate the score of concept drift for each unknown email to help our spam filtering method in judging the occurrence of concept drift. (3) We designed an incremental learning mechanism for our filtering method to strengthen its ability of adapting to the dynamic environment.

According to experimental results, the Precision, Recall, and F-measure of our spam filtering method would reach 96.0%, 99.5%, and 97.7%. With the incremental learning mechanism, obviously, the values of Precision, Recall and F-measure are increasing month after month. This means that, the weighted Sliding Window and email header analysis method can filter spam effectively without consuming too many system resources and calculation costs.
